# Five zinc finger protein 350 single nucleotide polymorphisms and the risks of breast cancer: a meta-analysis

**DOI:** 10.18632/oncotarget.21620

**Published:** 2017-10-07

**Authors:** Yu Fan Zeng, Jianfeng Sang

**Affiliations:** ^1^ From the Bachelor of Health Sciences, McMaster University, Hamilton, Ontario, Canada; ^2^ Department of General Surgery, Nanjing Drum Tower Hospital, Nanjing, China

**Keywords:** breast cancer, meta-analysis, SNP, ZNF350, DNA damage

## Abstract

Some studies have reported an association between the zinc-finger protein 350 (ZNF350), also known as zinc-finger and BRCA1-interacting protein with a Kruppel-associated box (KRAB) domain (ZBRK1), and risks of breast cancer, although the results remain controversial. A systematic search was conducted on PubMed, Web of Science, EMBASE, Ovid, Chinese National Knowledge Databases, and WanFang databases with relevant keywords. Four studies of five distinct populations involving 5824 breast cancer cases were used to conduct a meta-analysis that summarizes the current evidence of 5 genetic polymorphisms: Asp35Asp, Leu66Pro, Pro373Pro, Ser472Pro, and Ser501Arg in the ZNF350 gene. The T allele in Asp35Asp polymorphisms not significantly associated with increased risk of breast cancer (OR: 1.08; 95% CI: 0.96–1.21). The minor C allele of the Asp35Asp polymorphism is protective in the overdominant model (OR = 1.14; 95% CI: 1.02–1.28). The Pro allele in the Leu66Pro polymorphism is protective in all of the models examined (allelic, dominant, recessive, and overdominant). The Pro373Pro is not associated with breast cancer in all of the models tested. The Pro allele of the Ser472Pro polymorphism is protective using the dominant model (OR = 0.10; 95% CI: 0.04–0.23) but deleterious using the overdominant model (OR = 1.14; 95% CI: 1.02–1.28). The Ser501Arg polymorphism is deleterious only when using the recessive model (OR = 1.21; 95% CI: 1.02–1.44). In conclusion, this meta-analysis suggests that genetic polymorphisms in the *ZNF350* variant can increase, decrease, or have no effect on the risks of breast cancer depending on the polymorphism and genetic model used. Further studies will be required to validate these findings.

## INTRODUCTION

Breast cancer is the most common malignancy in women worldwide [1] and leads to 15% of all cancer-related deaths in women [2]. In western countries, the lifetime risk of developing breast cancer can be as high as 1 in 8 in women [3], resulting in a substantial burden to global health care.

Although the exact pathogenic mechanism of breast cancer remains elusive, polygenic models assuming a multiplicative effect on suggest that near 50% if all breast cancer cases fall within 12% of the population with increased risks of breast cancer [4, 5]. Thus, investigating the genetic factors playing roles in the development of breast cancer could not only aid in the search for pathological mechanism, but also have important influences on breast cancer screening and prevention.

Mutations in the tumour suppressor gene breast cancer 1 (BRCA1) accounts for almost 50% hereditary breast cancer [6] and reduced *BRCA1* expression strongly correlated with accelerate growth and progression of sporadic breast cancer [7]. *BRCA1* encodes a 220 kDa nuclear protein that is heavily involved in DNA damage repair, transcriptional regulation, and cell cycle checkpoint [8, 9]. Mutations in BRCA1 often leads to genomic instability [10]. It has been shown that BRCA1 recruits the DNA/RNA helicase Senataxin to resolve R-loops often found at transcriptional terminators and prevents R-loop accumulation-driven DNA damage [11].

The C-terminus of BRCA1 acts as an transcriptional activator to cell-cycle regulated genes such as growth arrest and DNA damage gene 45 (*GADD45*) and as also as GADD45A’s transcriptional corepressor during cell cycle G2/M checkpoint in association with the zinc-finger protein 350 (ZNF350) [12, 13]. ZNF350, also known as zinc-finger and BRCA1-interacting protein with a Kruppel-associated box (KRAB) domain (ZBRK1), have been shown to be involved in the pathogenic developments of several human tumours, such as breast, colon, and cervical carcinogenesis [14–16]. In cervical cancer, increased *ZNF350* gene expression is correlated with inhibition of growth and metastasis of cervical tumour cells, suggesting that ZNF350 could possibly be a tumour suppressor. A possible explanation is that the complex CtIP(CtTB interacting protein)/ZNF350/BRCA1 complex represses the expression of angiopoitin-1 (ANG1) and high-mobility group AT-hook 2 (HMGA2), which are commonly involved in the proliferation and vascular formation of breast tumours [17, 18]. ZN350 has been found to be a transcriptional repressor of p21 when associated with the KRAB domain-associated protein 1 and an increase of ZNF350 levels could result in sensitivity to DNA damage, possibly leading to carcinogenesis [19, 20].

In light of the recent evidence that ZNF350 gene variations is highly linked with breast cancer susceptibility and many previous studies have yielded conflicting results, we have conducted a thorough systematic review and meta analysis of the recent *ZNF350* risk alleles and their associations with breast cancer.

## RESULTS

### Study characteristics

**Figure 1:**
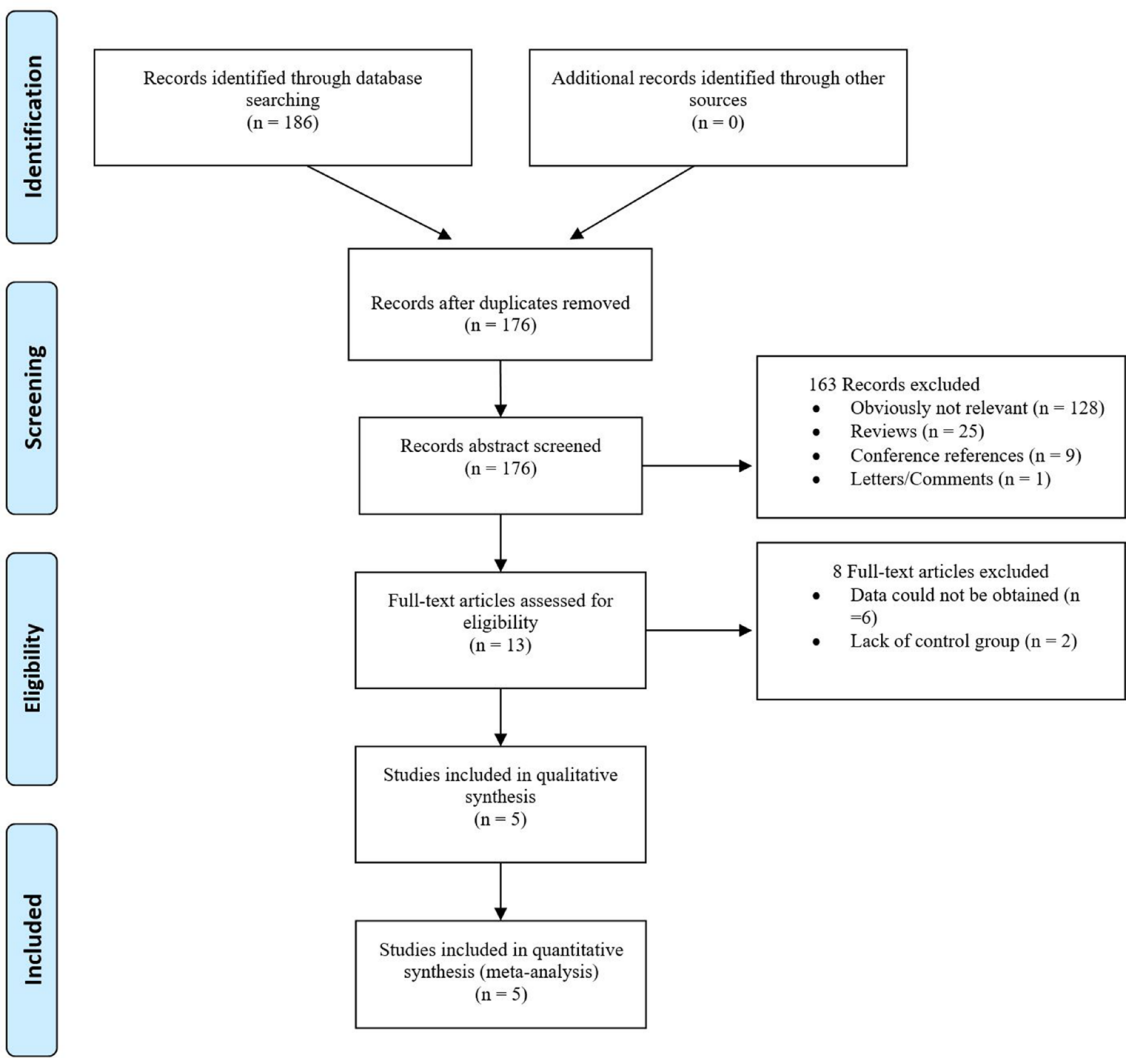
The PRISMA study selection diagram

The search yielded a combined 176 references. Study selection process was shown in Figure 1. The final meta-analysis included a total of 4 articles of 5 data sets [14, 21–23]. The 5 data sets included 6032 controls and 5824 breast cancer cases. The detailed characteristics of included studies are shown in Table 1.

**Table 1:** Characteristics of included studies

Study	Year of publication	Genotyping Method	Control	Average age (Case/Control)	Number of (case/Control)	Newcastle-Ottawa
Garcia	2004	Sequencing	Healthy Control	NA/NA	61/15	6
Garcia-Closas (Polish)	2006	Sequencing	Healthy Control	54/NA	1978/2283	7
Garcia-Closas (US)	2006	Sequencing	Healthy Control	56/NA	3181/3031	7
Desjardin [19]	2008	Sequencing	Healthy Control	48/46	97/94	6
Huo	2008	PCR-RFLP	Healthy Control	52/51	568/624	6

### Meta-analysis results

**Figure 2:**
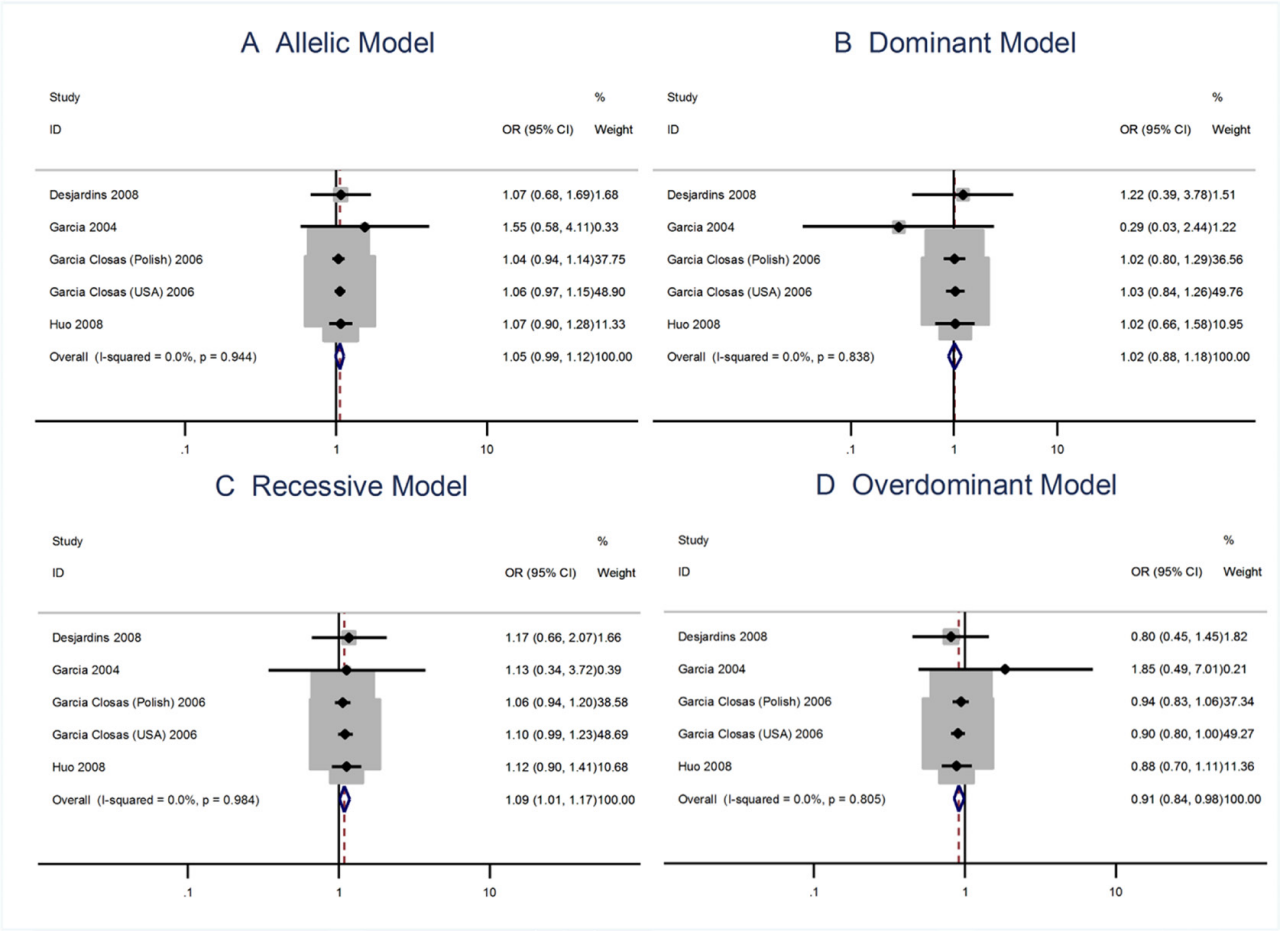
Meta analysis of the association between the Asp35Asp polymorphism and the risks of breast cancer using the (**A)** allelic, (**B**) dominant, (**C**) recessive, or (**D**) overdominant model.

The main results of the meta-analysis were listed in Table 2. Overall there was no evidence of an association between the risk allele C and increased risks of breast cancer when four data sets were pooled together for the Asp35Asp (T > C; rs4986773) polymorphism. The per-allele OR of Pro using the random effects models was 1.06 [95% CI: 0.99–1.15; P(Z) = 0.092; P(Q) = 0.944; Figure 2], although the data pooled OR was very close to being statistically significant. The recessive model does show that there is an association between the homozygous recessive and heterozygous and the risks of breast cancer [OR = 1.09; 95% CI: 1.01–1.17].

**Table 2:** Meta-analysis results

Polymorphism	Genetic Model	Number of data sets	Number of cases/controls	Minor Allele		
				OR (95% CI)	P (Z)	P (Q)
Asp35Asp (T > C);		5	5620/5519			
	Allelic			1.06 (0.99–1.15)	0.092	0.944
	Dominant			1.02 (0.88–1.18)	0.814	0.838
	Recessive			1.09 (1.01–1.17)	0.026	0.984
	Overdominant			0.91 (0.83–0.99)	0.036	0.986
Leu66Pro (A > G)		4	5824/6032			
	Allelic			0.84 (0.78–0.89)	0.000	0.000
	Dominant			0.57 (0.47–0.69)	0.000	0.002
	Recessive			0.86 (0.80–0.93)	0.000	0.000
	Overdominant			0.94 (0.82–1.0)	0.146	0.010
Pro373Pro (C > A);		3	5218/5060			
	Allelic			0.98 (0.90–1.06)	0.573	0.304
	Dominant			0.90 (0.68–1.18)	0.449	0.893
	Recessive			0.98 (0.90–1.07)	0.699	0.215
	Overdominant			0.99 (0.91–1.09)	0.884	0.180
Ser472Pro (C > A)		3	5041/4917			
	Allelic			0.96 (0.87–1.06)	0.433	0.002
	Dominant			0.10 (0.04–0.23)	0.000	0.119
	Recessive			1.04(0.93–1.17)	0.480	0.014
	Overdominant			1.14 (1.02–1.28)	0.022	0.107
Ser501Arg (T > A)		2	665/718			
	Allelic			0.95 (0.79–1.15)	0.612	0.168
	Dominant			0.80 (0.44–1.44)	0.454	0.842
	Recessive			1.21 (1.02–1.44)	0.026	0.017
	Overdominant			1.00 (0.80–1.25)	0.977	0.079

**Figure 3:**
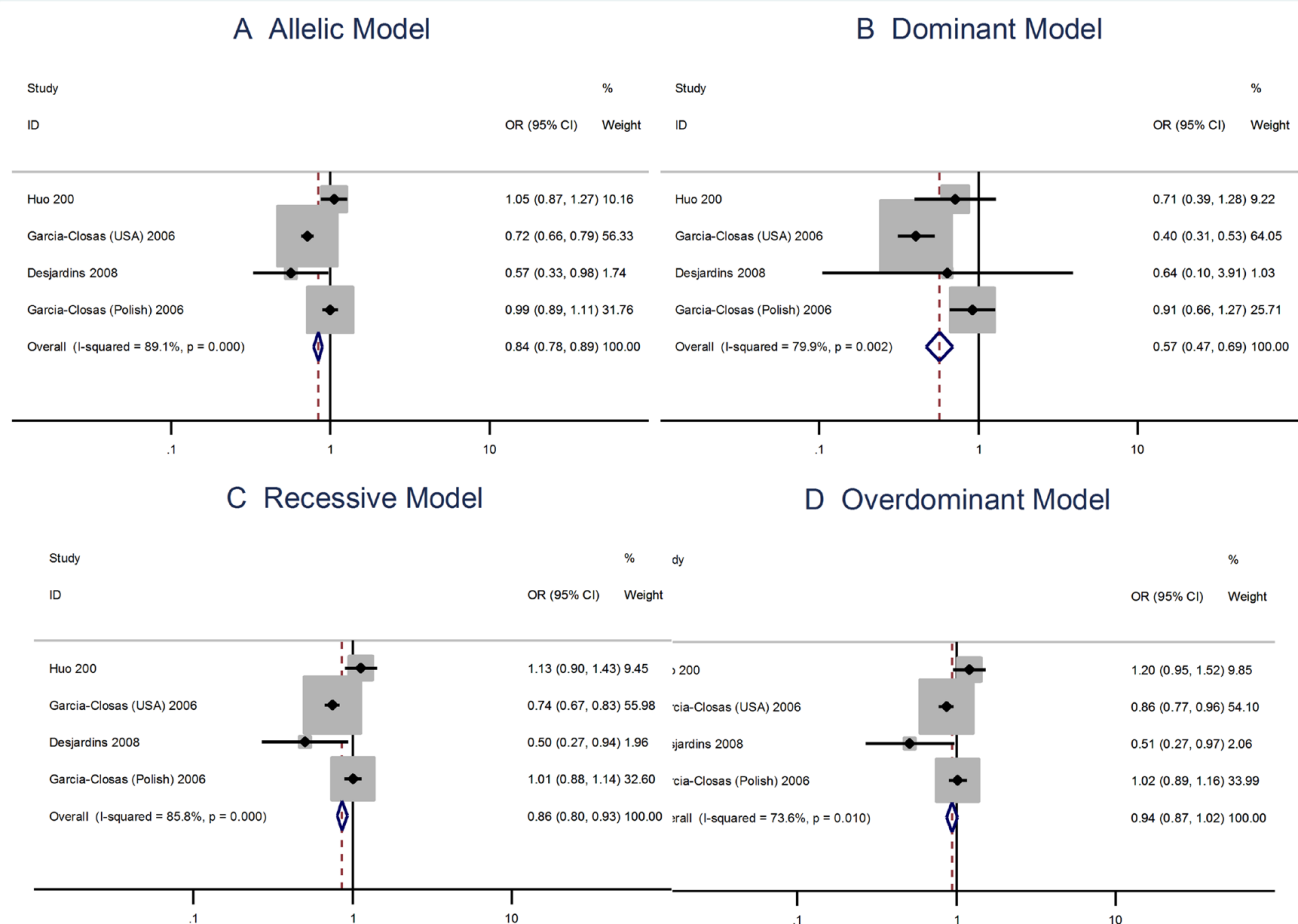
Meta analysis of the association between the Leu66Pro polymorphism and the risks of breast cancer using the (**A**) allelic, (**B**) dominant, (**C**) recessive, or (**D**) overdominant model.

The G allele in the Leu66Pro (A > G) polymorphism has a protective effect to the risks of breast cancer [OR = 0.84; 95% CI: 0.78–0.89]. The Leu66Pro polymorphism is also protective to the risks of breast cancer when using the the dominant, recessive, or the overdominant model (Figure 3).

**Figure 4:**
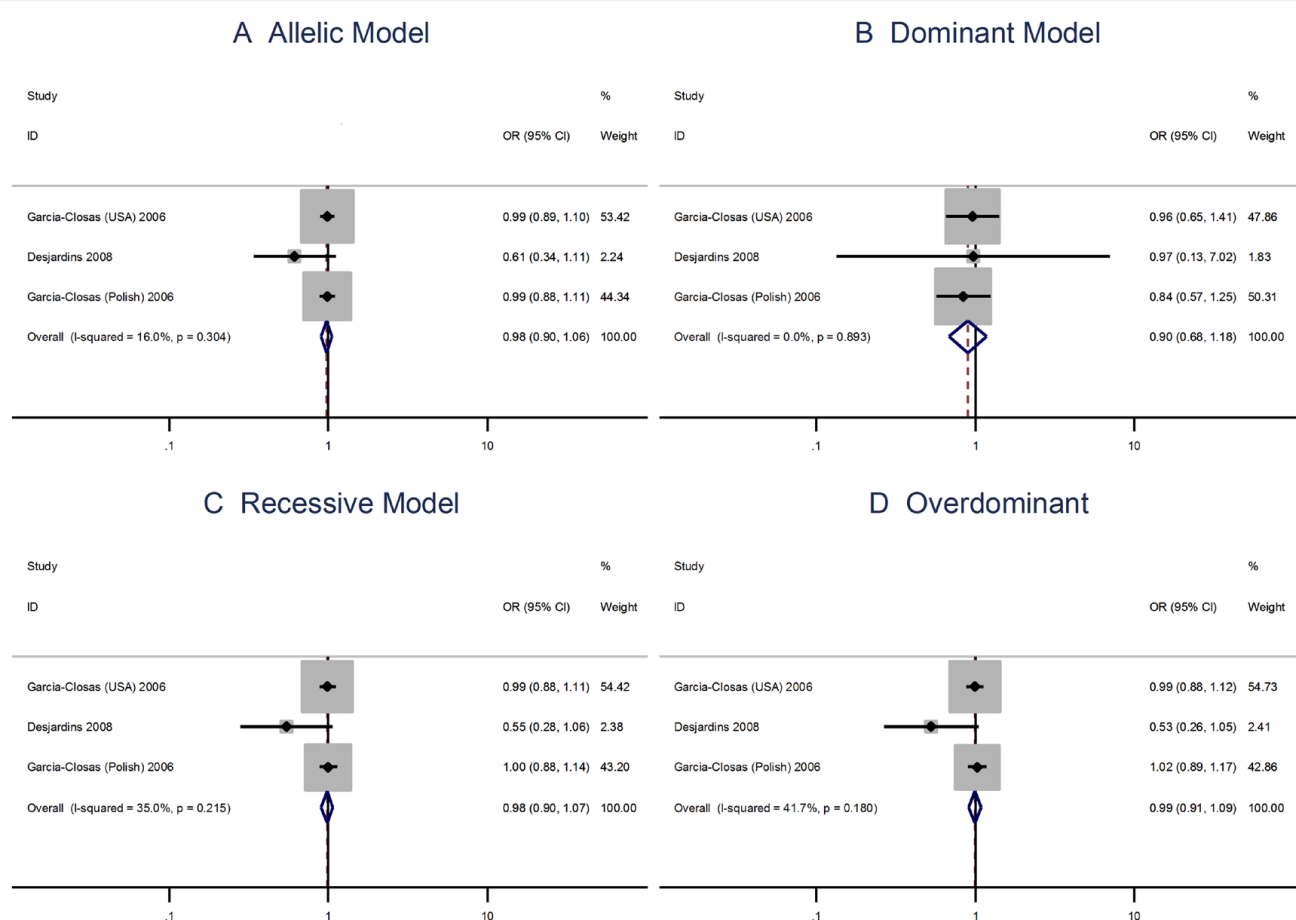
Meta analysis of the association between the Pro373Pro polymorphism and the risks of breast cancer using the (**A**) allelic, (**B**) dominant, (**C**) recessive, or (**D**) overdominant model.

**Figure 5:**
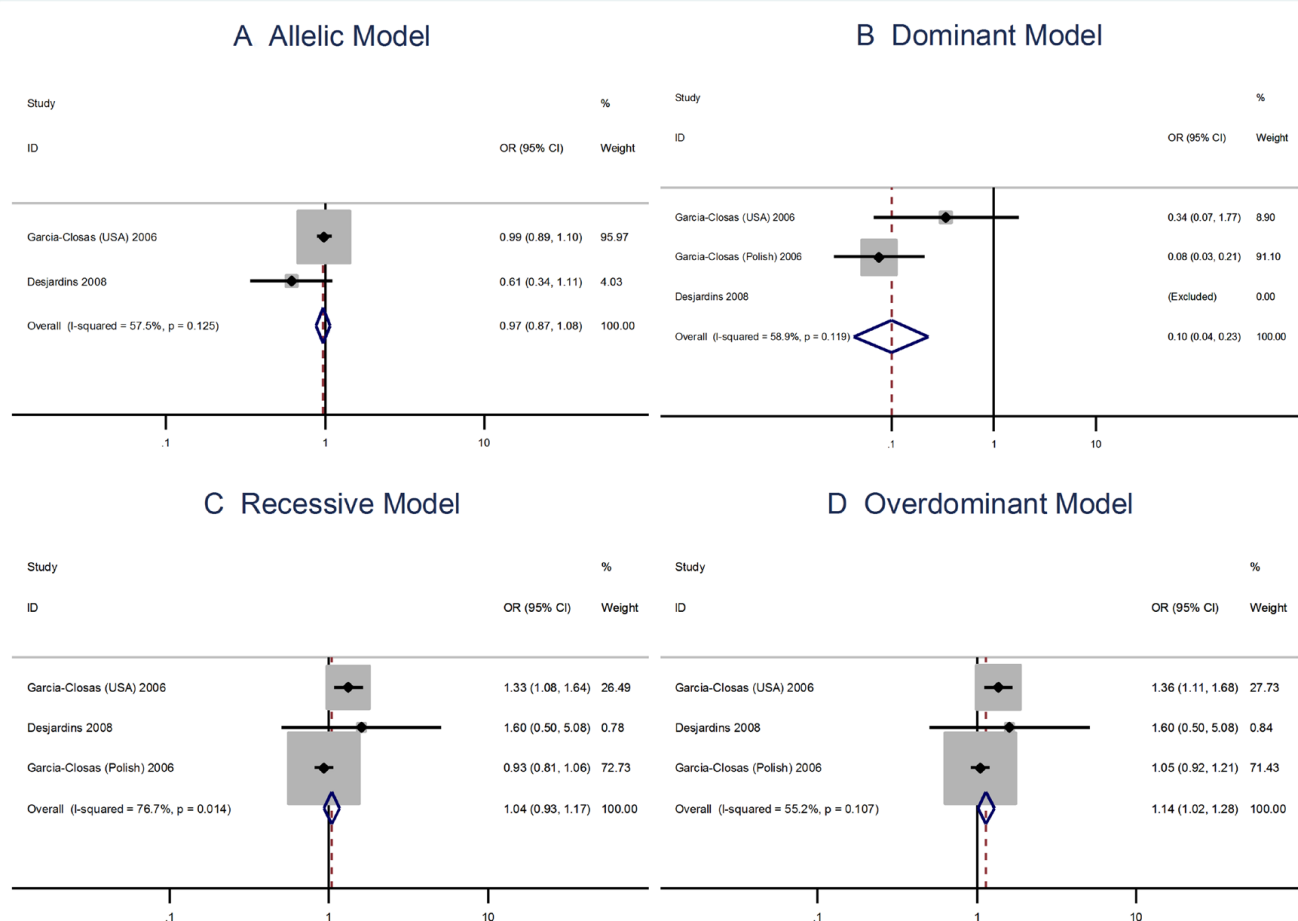
Meta analysis of the association between the Ser472Pro polymorphism and the risks of breast cancer using the (**A**) allelic, (**B**) dominant, (**C**) recessive, or (**D**) overdominant model.

The A allele Pro373Pro (C > A) polymorphism is not associated with risks of breast cancer using the allelic model (OR = 0.98; 95% CI: 0.90–1.06) or the dominant, recessive, or overdominant models (Figure 4). The Ser472Pro (C > A) polymorphism is no associated with the risks of vreat cancer using the allelic model (OR = 0.96; 95% CI: 0.87–1.06) or the recessive model (OR = 1.04; 95% CI = 0.93–1.17). However, the homozygous genotypes is associated with the risks of breast cancer using the overdominant model (OR = 1.14; 95% CI: 1.02–1.28; Figure 5).

**Figure 6:**
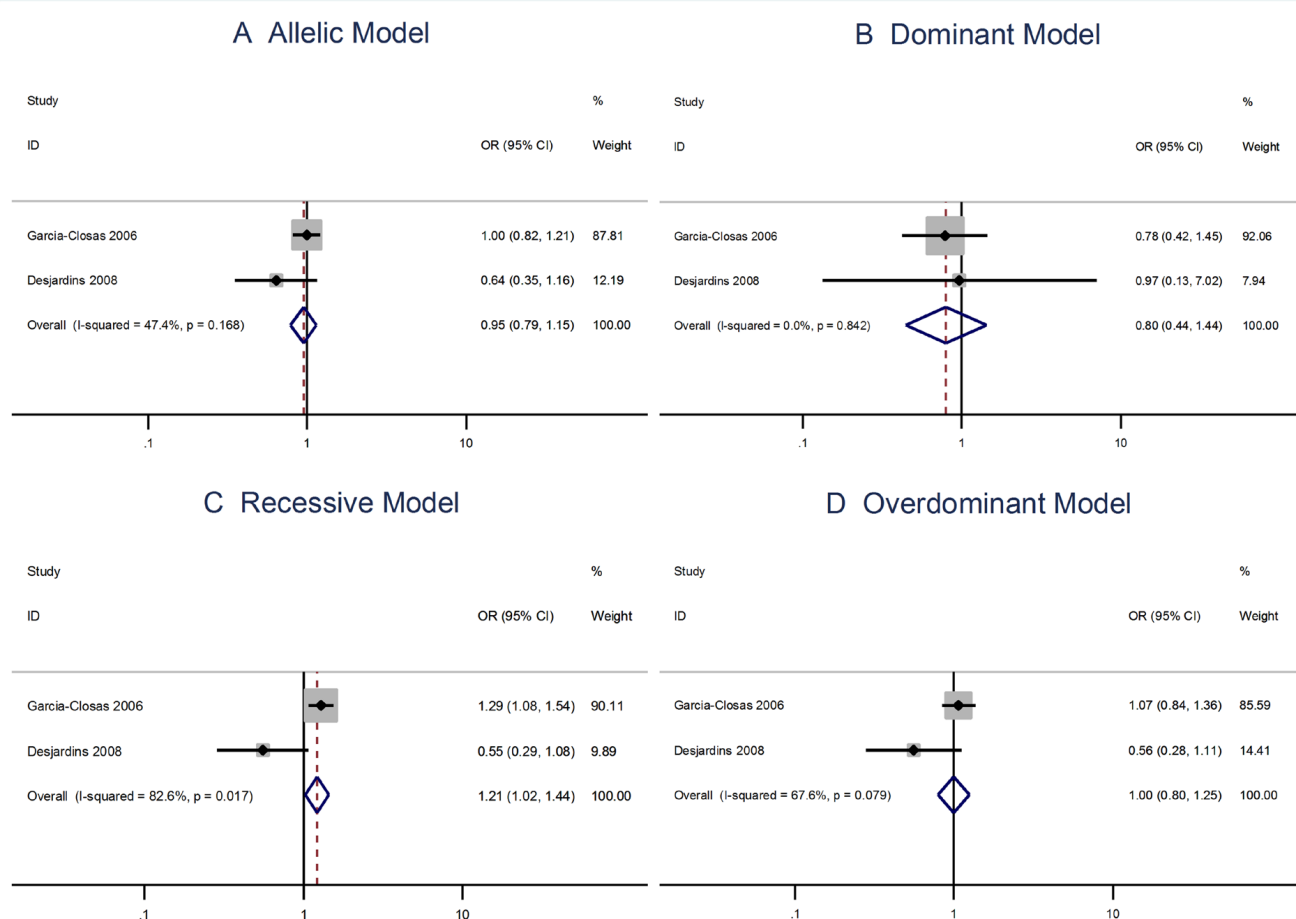
Meta analysis of the association between the Ser501Arg polymorphism and the risks of breast cancer using the (**A**) allelic, (**B**) dominant, (**C**) recessive, or (**D**) overdominant model.

The Arg allele of the Ser501Arg polymorphism is not associated with the breast cancer risks when using the allelic model (OR = 0.95; 95% CI: 0.79–1.15). However, the Ser501Arg polymorphism is linked to breast cancer when applying the recessive model (OR = 1.21; 95% CI: 1.02–1.44; Figure 6).

### Sensitivity analysis

Sensitivity analyses using single-study omission demonstrated that this meta-analysis was stable. Statistical significance of the summary ORs was not modified. The data is not shown due to that the number of studies included is too low for some of the polymorphisms. However, a cumulative meta-analysis also shows that the results of this study are stable.

### Publication bias

**Figure 7:**
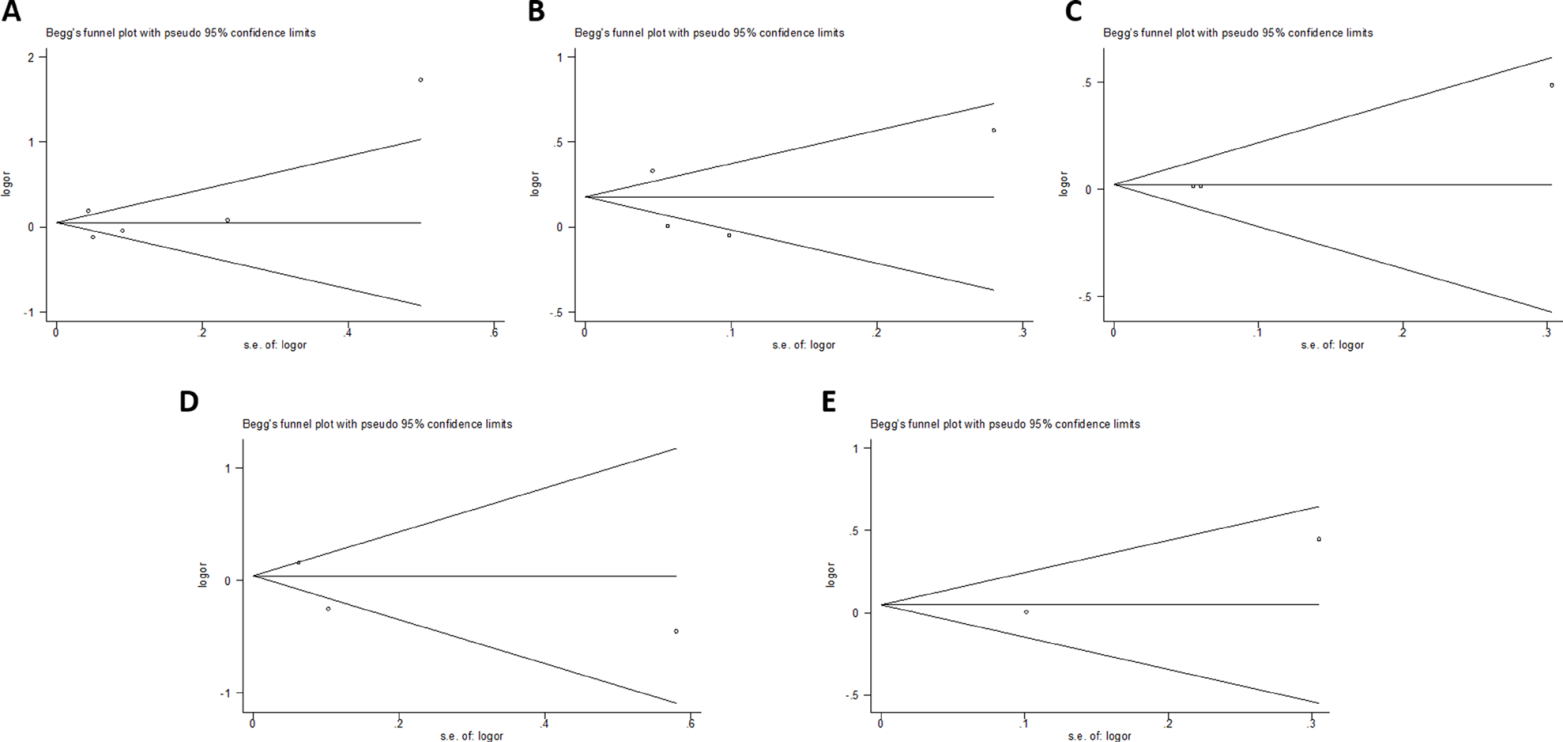
Begg’s plot of the included studies for the (**A**) Asp35Asp, (**B**) Leu66Pro, (**C**) Pro373Pro, (**D**) Ser472Pro, and (**E**) Ser501Arg polymorphisms.

**Figure 8:**
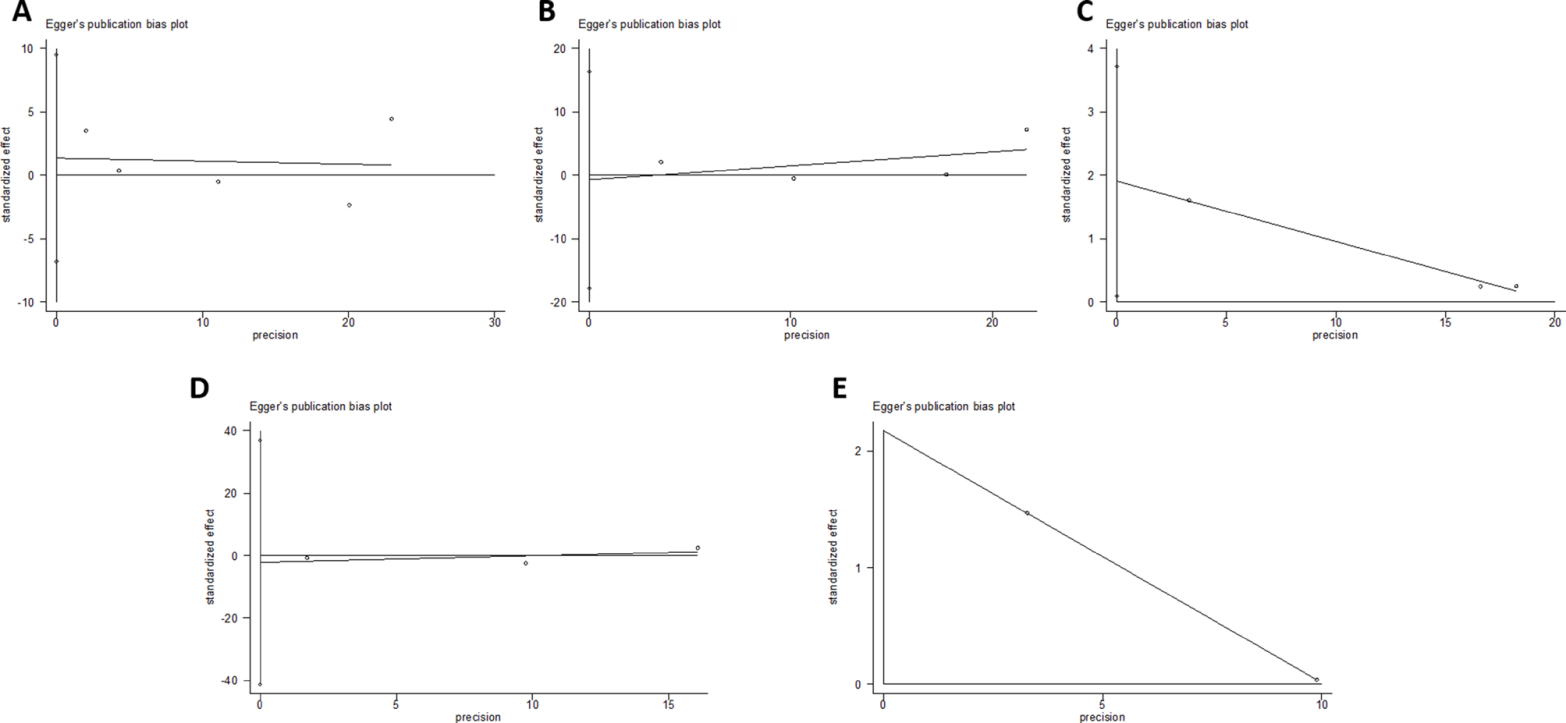
Egger’s plot of the included studies for the (**A**) Asp35Asp, (**B**) Leu66Pro, (**C**) Pro373Pro, (**D**) Ser472Pro, and (**E**) Ser501Arg polymorphisms.

Begger’s and Eggar’s funnel plots were constructed using the standard error and compared against the OR of each study (Figure 7 and Figure 8). The plots do not suggest the existence of publication bias towards positive findings in smaller studies. Further, the Duval and Tweedie nonparametric “trim and fill” method was utilized to adjust for publication bias and its results did not show different conclusions (data not shown) [24]. Thus, this indicates that this meta-analysis is statistically robust.

## DISCUSSION

Many genes involved in DNA repair and cell cycle have been linked with breast cancer [25–27], including the genes BRCA1 and BRCA2 which direct DNA double-stranded break by homologous recombination [28, 29]. ZNF350 is a transcriptional repressor that has been suggested as a tumor suppressor due to its close corporation with BRCA1 and KAP-1 to silence DNA damage response genes.

ZNF350 is a 532 amino acid protein that contains an N-terminal A+B box domain, eight C_2_H_2_ zinc fingers in the centre, and a C-terminal repression domain (CTRD) [13, 30]. The CTRD undergoes tetrameric oligomerization that allows ZNF350 to selectively interact with BRCA1, specific histone deacetylases, and specific promoters [31, 32]. ZNF has been found to repress the transcription of genes such as *GADD45A* [13], *ANG1* [18], *HMGA1* [17], *p21* [20], *MMP9* [16], *FGF2* [33], and *KAP1* [31].

The ZNF350 gene is mapped to chromosome 19q13.4 and is near a cluster of other KRAB-Zinc finger proteins. SNPs in ZNF50 has been previously linked to the risks of developing breast cancer, although to conflicting results [14, 21–23].

We have conducted the first meta-analysis summarizing the evidence regarding the association between the five ZNF350 SNPs and the risks of developing breast cancer. Our results suggest that the Leu66Pro polymorphism is mostly likely associated with modifying the risks of breast cancer, while patients with Asp35Asp and Ser501Arg SNPs have increased odds of breast cancer. Asp35Asp and Ser472Pro could be protective using the overdominant model.

Publication bias, the preferential publication of studies with positive results, is a significant problem in many meta-analyses. However, our meta-analysis also includes studies with negative conclusions. Furthermore, our funnel plots do not appear asymmetric, suggesting that publication bias is not a problem within our study.

Several limitations should be noted in interpreting the results of our meta-analysis. First, we were not able to adjust for potential confounding effects conferred by age, gender, lifestyle, and environmental factors due to the lack of individual data. Our results were based on unadjusted estimates - a more precise analysis could be conducted if all raw data related to the confounders were available. The lack of important individual health data, such as body mass index (BMI), drug history, diet, and age, also forbid us from performing a more sensitive analysis.

Although the functional significances of the mutations studied in the current study is unknown, we postulate that mutations such as Leu66Pro have many important biological roles as it is found in the KRAB-domain of the protein. In conclusion, the pooled results of our meta-analysis studying We propose to that both future large-scale clinical studies and functional studies are needed to elucidate the role to which ZNF350 modify the risks of breast cancer.

## MATERIALS AND METHODS

### Search strategy and inclusion criteria

We searched the literature hosted on PubMed, Web of Science, EMBASE, Ovid, Chinese National Knowledge Databases and WanFang with keywords related to breast cancer and the gene of interest (e.g. “zinc finger 350”, “ZNF350”, “zinc-finger and BRCA1-interacting protein with a KRAB domain 1”, and “ZBRK1”). Genetic association studies published before May 2017 were retrieved and no earlier publication date limit was applied. The last search was performed on the May 1st, 2017. For each study screened, we checked their references to identify other relevant publications to the topic. The systematic search was conducted without any restrictions on the language used and the minimum number of patients required to be included. The study focused on human studies.

All retrieved study were screened and considered elgible if satisfying each point of the following criteria: 1) case-control or cohort study, 2) original papers containing independent trials, 3) confirmation of breast cancer, and 4) genotype distribution information of both the control and experimental group or odds ratio (OR) with its 95% confidence interval (CI) and P value. The major reasons for exclusion of studies were case-only studies, overlapping data, insufficient data for analyses, and review articles.

### Data extraction

Data extraction was performed independently by two reviewers using a standard extraction form. All data were checked for internal consistency and disagreements were resolved through thorough discussion between all authors. If there were doubts about the result of studies, the corresponding author of the study of interest was contacted. For each study, the following were extracted from each article: first author’s name, publication year, diagnostic criterion, definition and numbers of cases and controls, ethnicity of the study population, frequency of genotypes, genotyping method, source of controls, Hardy–Weinberg equilibrium (HWE), age, body-mass index (BMI). Studies with different ethnic groups within the same study were considered as individual studies for our analyses.

### Statistical analysis

The association strength between the five ZNF350 polymorphisms and breast cancer was assessed by calculating OR with 95% CI.

The chi-square (χ^2^) test was used to evaluate whether there is a significant deviation from HWE among the control subjects of the study. The per-allele OR of risk allele T was compared between cases and controls in each study. The ORs were pooled using both the random-effects model (the DerSimonian and Laird method) and the fixed effects model (the Mantel-Haenszel method) as previously described [34, 35]. The Woolf’s method was used to calculate 95% CI [36]. The results of calculations using the random effects model were reported in this article because it takes into consideration the variation between studies.

Heterogeneity across individual studies was examined using Cochran’s χ^2^ *Q* test [37]. *Q* test was also performed to detect the heterogeneity within each subgroup. Publication bias was assessed using linear regression to measure funnel plot asymmetry on the natural logarithm of OR using Egger’s method [38]. All statistical analysis was carried out with Stata Version 13.0 (Stata Corporation, College Station, Texas, USA. All *P* values were for two-sided analysis. Type I error rate was set at 0.05.

## Abbreviations

ANG1: angiopoitin-1
BRCA1: breast cancer 1
BMI: body mass index
CI: confidence Interval
CtIP: CtTB interacting protein
GADD45A: growth arrest and DNA damage gene 45
HWE: Hardy–Weinberg equilibrium
KRAB: Kruppel-associated box
OR: odds ratio
ZNF350: zinc finger 350
ZBRK1: zinc-finger and BRCA1-interacting protein with a KRAB domain 1
